# The Chinese version of the general benefit finding scale (GBFS): Psychometric properties in a sample of college students

**DOI:** 10.1371/journal.pone.0300064

**Published:** 2024-05-07

**Authors:** Zhaozhao Hui, Xuan Wang, Ziyi Teng, Wenfeng Zou, Jing Wang, Pengcheng Ji, Mingxu Wang

**Affiliations:** 1 School of Public Health, Xi’an Jiaotong University Health Science Center, Xi’an, Shaanxi, China; 2 Health Culture Research Center, Key Research Base of Philosophy and Social Sciences in Shaanxi, Xianyang, Shaanxi, China; 3 Tongchuan Peolple’s Hospital, Tongchuan, Shaanxi, China; 4 Zonglian College, Xi’an Jiaotong University, Xi’an, Shaanxi, China; 5 Periodicals Publishing House, Xi’an Jiaotong University, Xi’an, Shaanxi, China; Aalborg University, DENMARK

## Abstract

**Background:**

Benefit finding has become a central construct in the evolution of positive psychology and attracted attention in recent literature. This study aimed to translate and validate the General Benefit Finding Scale (GBFS) in Chinese college students.

**Methods:**

Forward- and back-translation of the GBFS was followed by the assessment of semantic equivalence and content validity. A sample of 589 college students was recruited in China to conduct reliability and validity analysis. The construct validity was assessed using exploratory (EFA) and confirmatory factor analysis (CFA). Concurrent validity was assessed using Pearson’s correlation coefficients of the GBFS with the Perceived Stress Scale (PSS) and World Health Organization-Five Well-Being (WHO-5). Internal consistency and two-week test-retest reliability were also evaluated.

**Results:**

The content validity index for each item ranged from 0.83 to 1.00. EFA revealed a six-factor model, which exhibited acceptable goodness of fit in CFA (standardized root mean square residual = 0.031, root mean square error of approximation = 0.059, goodness-of-fit index = 0.860, comparative fit index = 0.904, Tucker-Lewis index = 0.890, chi-squared/degree of freedom *=* 2.07). The concurrent validity of the GBFS was supported by its statistically significant correlations with PSS (*r* = –0.271, *p*<0.001) and WHO-5 (*r* = 0.354, *p*<0.001). Moreover, the internal consistency for the overall scale was satisfactory, with Cronbach’s α coefficient of 0.93 and McDonald’s omega reliability of 0.94. The test-retest reliability was 0.82.

**Conclusions:**

Although the Chinese version of GBFS was examined in a homogeneous convenience sample of college students, it provides a reliable and valid instrument for assessing benefit finding in the Chinese context.

## Introduction

Benefit finding refers to a reported positive life change resulting from the struggle to cope with a challenging life event such as trauma, illness, or other negative experiences [1]. It is often used interchangeably with posttraumatic growth, adversarial growth, and stress-related growth in literature [2]. Evidence has accumulated regarding the co-occurrence of positive and negative emotions during stressful periods [3] and there is increasing recognition that some individuals appraise their negative experiences as beneficial [4]. Benefit finding could be maladaptive if it impedes important problem-focused coping (e.g., information gathering and evaluating) [3]. However, most studies have shown that benefit finding leads to better psychological and physical health outcomes in various populations [5–7]. For example, a recent systematic review found that benefit finding was negatively associated with anxiety, depression, and distress in cancer patients [5]. A double-blind randomized controlled trial revealed that benefit finding could significantly reduce depressive symptoms and role overload in Alzheimer caregivers [6]. It has been proposed that benefit finding may involve changes in psychological domains that lead to a state of enhanced allostasis, buffering against negative effects of catabolic stress responses and promoting activity in restorative physiological systems [7].

While benefit finding becomes a central construct in the evolution of positive psychology [8], its measurement is receiving increasing attention from scholars. Pascoe and Edvardsson have identified 17 existing benefit finding instruments in cancer practice [9]. Of them, the Benefit Finding Scale (BFS), originally developed for women diagnosed with breast cancer [10], was the most widely adopted in research. This scale consists of 17 items that begin with “having had breast cancer” [10]. The BFS has been translated into several languages (e.g., German, Spanish) and its psychometric properties have been examined in other cancer patients [11, 12]. With increasing interest in benefit finding, a growing number of instruments were developed for different populations [13–19], including children with cancer [13], people with chronic conditions [14–16], and caregivers [17–19] (Table 1). Despite this, measures of benefit finding have tended to be domain-specific.

**Table 1:** Examples of benefit finding measurement.

Scale	Items	Apply for
1. Benefit Finding Scale [10]	17	Breast cancer patients
2. Benefit Finding Scale for Children [13]	10	Children with cancer
3. Benefit Finding Questionnaire [14]	21	People with mental illness
4. Japanese Benefit Finding scale [15]	26	Patients with cancer
5. Benefit Finding in Multiple Sclerosis Caregiving Scale [16]	27	Persons with multiple sclerosis and their carers
6. Caregiver Benefit Finding Scale [17]	26	Family caregivers of stroke survivors
7. Diabetes Benefit Finding Scale for Parents [18]	16	Parents of young children with type 1 diabetes
8. Benefit Finding Scale for Parents of Children with Asperger Syndrome [19]	40	Parents of children with Asperger syndrome

Considering that individuals can encounter adversity in the events and hassles of everyday life, Cassidy et al. developed the General Benefit Finding Scale (GBFS) to ascertain levels of benefit finding in relation to general life stress as opposed to chronic illness or trauma [8]. The GBFS comprises 28 items with six dimensions: acceptance, family bonds, personal growth, relationships, empathy, and reprioritization [8, 20]. It has gradually been used to measure benefit finding in the field of general life stress. Zimmermann et al. assessed college students’ perceived benefits of the COVID-19 pandemic through the GBFS [21]. Tan et al. adopted this scale to evaluate benefit finding in caregivers of individuals with mental illness [22]. Moreover, the GBFS has been translated into German and was used in adolescents to measure their subjective experience of positive changes in response to lifetime adversity [23, 24].

In China, the 17-item BFS, which was suitable for assessing benefit finding levels of cancer patients, has been translated and validated [25]. Mei et al. have recently developed a Caregiver Benefit Finding Scale specifically for stroke caregivers [17]. To our knowledge, the GBFS as a more generic benefit finding scale has not yet been validated in the Chinese population. Given the cultural differences [26, 27], we translated the GBFS into simplified Chinese with authorization from the original authors. This study aimed to examine the reliability and validity of GBFS in Chinese college students, which would provide evidence for further research such as developing tailored interventions for this population. It was hypothesized that the Chinese version of GBFS would demonstrate satisfactory internal consistency, test-retest reliability, content validity, and construct validity. According to Folkman, benefit finding is an important meaning-focused coping strategy [3], which may contribute to alleviating stress [28] and promoting well-being [29] during a stressful period. We thus hypothesized that the GBFS score would significantly correlate with perceived stress and subjective well-being in Chinese college students, which suggests good concurrent reliability.

## Materials and methods

### Procedure

#### Translation process

The scale was translated into simplified Chinese according to the forward-backward translation method, following the guidelines published by Sousa et al. [30]. With permission from the author of the original scale, an assistant professor and a postgraduate, fluent in English and Chinese, performed the forward translation. Any discrepancies and ambiguities between the two forward-translated versions were discussed and resolved by discussion with a third translator. Through this process, we generated a preliminary initial Chinese version of GBFS, which was then translated back into English by two postgraduates independently. Any discrepancies and ambiguities between the two back-translations and between each of the back-translations and the original English version were discussed and resolved to derive a pre-final version of the Chinese version of GBFS.

#### Evaluation of semantic equivalence

The pre-final version of the Chinese version of GBFS was provided to an expert panel of five researchers to determine the content equivalence. The equivalence of the instruction, response format, and each item of the scale was evaluated on a four-point Likert scale ranging from 1 (least appropriate) to 4 (most appropriate). The researchers were advised to provide comments on those item(s) that they gave ratings of 1 or 2. Items classified as 1 or 2 were then revised accordingly.

#### Psychometric testing

To evaluate the content validity, the GBFS was provided to an expert panel, which consisted of a professor, an assistant professor, and four doctoral students. Those experts rated the degree of relevance of each item to the construct they intended to measure (i.e., benefit finding) on a 4-point Likert scale ranging from 1 (least relevant) to 4 (highly relevant) [31] (phase 1).

Additionally, the GBFS was distributed to a sample of college students, and exploratory factor analysis (EFA) was performed to identify the possible components of the GBFS in Chinese (phase 2). A cross-sectional study was then conducted in another sample of college students to examine the internal consistency, construct validity (confirmatory factor analysis [CFA]), and concurrent validity of the scale (phase 3). After two weeks, 50 college students who had completed the questionnaires in phase 3 were surveyed again to assess the test-retest reliability [32] (phase 4).

### Participants

College students were recruited from Xi’an, Shaanxi, China in March 2022, by convenience sampling methods. The inclusion criteria for the participants were as follows: (1) Chinese citizens aged 18 years or older; (2) full-time college students; and (3) be able to give written consent. The students who had experienced major life events (e.g., death of a loved one) in the last three months or those diagnosed with a mental disorder were excluded from this study. The sample size was estimated based on a widely accepted rule of thumb, in which 10 cases per variable are required for factor analysis [33]. The GBFS consists of 28 items, 280 participants therefore should be included. This study was approved by the Biomedical Ethics Committee of Xi’an Jiaotong University Health Science Center (2022–0005). Prior to data collection, written informed consent was obtained from all participants.

### Measurements

#### Socio-demographic questionnaire

Socio-demographic characteristics of the college students were collected via a questionnaire developed by the research team. The socio-demographic data included age (years), gender (male or female), and grade (freshman, sophomore, junior, senior).

#### General benefit finding scale (GBFS)

The GBFS consists of 28 items with six dimensions: acceptance (items 1–5), family bonds (items 6–9), growth (items 10–15), relationships (items 16–19), empathy (items 20–24), and reprioritization (items 25–28) [8]. The participants were asked to consider difficult times they have had in their lives and to answer each item on a 5-point Likert scale from 1 (strongly disagree) to 5 (strongly agree). The total score of GBFS was derived from summing up the responses of all items (ranges: 28–140). A higher score indicates a higher level of benefit finding (S1 File).

#### Perceived stress scale (PSS)

To evaluate the concurrent validity of GBFS, perceived stress was measured by the 10-item Chinese version of PSS [34]. The participants answered each item from 0 (never) to 4 (very often) in relation to the last month. The total score of PSS-10 was obtained by reversing the scores on the positive items (items 4, 5, 7, & 8) and then summing across all the items (ranges: 0–40). The higher the score, the more perceived stress. The PSS-10 has been shown to have good reliability and validity in both adult and university student populations [35]. In the current study, the Cronbach’s α coefficient for the PSS-10 was 0.723.

#### World health organization-five well-being index (WHO-5)

Subjective well-being was also measured to evaluate the concurrent validity of GBFS, by using the Chinese version of WHO-5 [36]. The participants responded to each item from 0 (at no time) to 5 (all the time) and the raw score was calculated by summing up the five answers (ranges: 0–25). To obtain a final score, the raw score was multiplied by 4, ranging from 0 to 100, with 0 representing the worst subjective well-being and 100 representing the best. It has been demonstrated that the WHO-5 has appropriate reliability and validity and has been applied successfully across a wide range of study fields [37]. In the current study, the Cronbach’s α coefficient for the WHO-5 was 0.913.

### Data analyses

Statistical analyses were performed by the Statistical Package for Social Sciences (SPSS) version 25.0, AMOS 26.0 statistical software (IBM Corp., Armonk, NY, USA), and psych package in R 4.2.2. Descriptive analyses were conducted to summarize the variables in this study and Pearson correlation analysis was performed to analyze the inter-correlations between each item and the total score of GBFS. The correlation coefficient (*r*)<0.40 was considered weak, 0.40≤*r*<0.70 moderate, and *r*≥0.70 strong [38].

Reliability included internal consistency and test-retest reliability. Cronbach’s α coefficients for the overall GBFS and for each dimension, composite reliability index, as well as McDonald’s omega coefficient, were calculated to estimate the internal consistency, while test-retest reliability was determined by calculating the correlation coefficient (Pearson correlation analysis) and by analyzing the statistical differences in GBFS scores (paired sample *t*-test), with a random sample of 50 college students who completed the survey twice at a two-week interval. These coefficients higher than 0.7 were considered desirable [38–40].

Content validity, construct validity, and concurrent validity were used to assess the validity. Content validity was evaluated by the content validity index for each item (I-CVI) as well as the scale-level CVI based on the universal agreement (S-CVI/UA) and average method (S-CVI/Ave). For each item, the I-CVI was computed as the number of experts giving a rating of either 3 or 4, divided by the number of experts. S-CVI/UA was calculated by adding all items with I-CVI equal to 1 divided by the total number of items, while S-CVI/Ave was calculated by taking the sum of the I-CVIs divided by the total number of items [41]. Excellent content validity should be composed of I-CVIs of 0.78 or higher [42] and S-CVI/UA and S-CVI/Ave of 0.8 and 0.9 or higher, respectively [43].

Construct validity was evaluated by conducting EFA (principal component analysis) [44] and CFA [45]. When performing EFA, the Kaiser criterion (eigenvalue≥1.0), cumulative variance contribution rate, and scree plot were considered in the determination of factor solution. When the factor loading≥0.40, the item can be considered in the relevant factor [46]. In terms of CFA, the goodness of fit of the model was assessed by the following criteria: standardized root mean square residual (SRMR)<0.08, root mean square error of approximation (RMSEA)<0.06, goodness-of-fit index (GFI)>0.85, comparative fit index (CFI) and Tucker-Lewis index (TLI)>0.90, and chi-squared/degree of freedom (*χ*^2^/*df*)<5 indicated that the model fit was acceptable [45, 47].

The concurrent validity of the GBFS was assessed by its correlations with perceived stress (PSS) and subjective well-being (WHO-5), which were analyzed by Pearson correlation analysis. The significance level in this study was set at 0.05 (two-tailed).

## Results

### Participant characteristics

A total of 589 Chinese college students were included in the final data analysis of this study. In phase 2, we collected 289 questionnaires, of which 9 were considered as invalid since more than five items were missing (effective recovery rate: 96.9%). The remaining 280 participants aged 20.6 years (standard deviation [SD] = 1.4) were thus included for data analysis (i.e., EFA).

In phase 3, a total of 320 questionnaires were returned but 11 were eliminated due to missing values. Therefore, 309 participants were included for data analysis (effective recovery rate: 96.6%) (S2 File). They aged 20.5±1.5 years old and half of them (n = 168) were male. For the grade, 15.2%, 26.9%, 27.2%, and 30.7% were freshmen, sophomores, juniors, and seniors, respectively (Table 2).

**Table 2:** Characteristics of the participants.

Variables	Stages	
	Phase 2 (N = 280)	Phase 3 (N = 309)
Age (years, M±SD)	20.6±1.4	20.5±1.5
Gender		
Male	153 (54.6)	168 (54.4)
Female	127 (45.4)	141 (45.6)
Grade		
Freshman	26 (9.3)	47 (15.2)
Sophomore	79 (28.2)	83 (26.9)
Junior	80 (28.6)	84 (27.2)
Senior	95(33.9)	95 (30.7)

Notes: M = mean, SD = standard deviation

### Item analysis

The descriptive statistics of the 28 items and their correlations with the total score of GBFS are demonstrated in Table 3. As seen, the skewness and kurtosis statistics of all items were within ±2.00 which indicates a normal distribution [48]. The correlation coefficient between each item and the total score ranged from 0.421 (item 27) to 0.691 (item 14) and was all statistically significant (*p*<0.001).

**Table 3:** Descriptive statistics and item total correlations (N = 309).

Items	M (SD)	Skewness	Kurtosis	*r*	Items	M (SD)	Skewness	Kurtosis	*r*
1	3.4 (1.01)	–0.414	–0.291	0.578***	15	4.0 (0.81)	–0.861	1.199	0.649***
2	3.8 (0.85)	–0.653	0.457	0.538***	16	3.8 (0.95)	–0.691	0.011	0.647***
3	3.6 (0.97)	–0.491	–0.280	0.564***	17	3.9 (0.85)	–0.566	0.242	0.567***
4	3.6 (0.96)	–0.636	0.099	0.561***	18	3.3 (1.01)	–0.231	–0.317	0.563***
5	3.8 (0.97)	–0.673	0.216	0.634***	19	3.6 (0.96)	–0.499	0.135	0.550***
6	3.6 (1.00)	–0.360	–0.405	0.544***	20	4.0 (0.85)	–0.981	1.44	0.595***
7	3.9 (0.90)	–0.660	0.254	0.639***	21	3.7 (0.98)	–0.571	0.033	0.497***
8	4.0 (0.89)	–0.680	0.035	0.589***	22	3.8 (0.90)	–0.419	–0.212	0.600***
9	4.0 (0.91)	–0.810	0.390	0.529***	23	4.0 (0.88)	–1.111	1.729	0.651***
10	3.5 (0.99)	–0.427	–0.284	0.672***	24	3.9 (0.88)	–0.881	0.993	0.650***
11	3.8 (0.88)	–0.685	0.439	0.630***	25	3.5 (1.05)	–0.339	–0.406	0.597***
12	3.9 (0.86)	–0.865	0.961	0.623***	26	3.2 (1.04)	–0.057	–0.622	0.470***
13	3.8 (0.97)	–0.704	0.259	0.673***	27	3.5 (0.99)	–0.346	–0.362	0.421***
14	3.8 (0.92)	–0.728	0.609	0.691***	28	3.9 (0.89)	–0.764	0.647	0.502***

Notes: M = mean, SD = standard deviation

*** *p<*0.001

### Validity

#### Content validity

Six experts were invited to rate content validity. Of them, two had doctoral degree (33.3%) and the others had master’s degree (66.7%). Results indicated satisfactory content validity of the Chinese version of GBFS. Specifically, the I-CVI ranged from 0.83 to 1.00, with an average of 0.99 (S-CVI/Ave). The proportion of items that obtained a relevance score of 3 or 4 from all experts (S-CVI/UA) was 0.96.

#### Construct validity

The Kaiser-Meyer-Olkin value was 0.93 and Bartlett’s test of sphericity reached statistically significant (*χ*^2^ = 3726.11, *df* = 378, *p*<0.001), thus indicating that the correlation matrix was suitable for factor analysis. Consistent with the original scale structure [8], the EFA results exhibited a six-factor model, namely acceptance (items 1–4), family bonds (items 6–9), growth (items 5 & 10–15), relationships (items 16–19), empathy (items 20–24), and reprioritization (items 25–28). The explained variances of the six factors were 7.4%, 10.6%, 15.9%, 9.9%, 9.5%, and 8.2%, respectively. The contribution rate was 61.5%, and the factor loadings of all items were between 0.446 and 0.814 (Table 4).

**Table 4:** Factor loadings for the GBFS items (N = 280).

Items	Factor 1	Factor 2	Factor 3	Factor 4	Factor 5	Factor 6
1. Led me to be more accepting of things	**0.625**	0.112	0.407	0.011	0.201	-0.036
2. Taught me how to adjust to things I cannot change	**0.446**	0.346	0.284	-0.091	0.245	0.193
3. Helped me take things as they come	**0.720**	0.083	-0.031	0.175	0.027	0.199
4. Given me a more realistic set of expectations	**0.652**	0.179	0.105	0.186	0.121	0.147
5. Taught me to be patient	0.286	0.324	**0.533**	0.084	0.087	0.126
6. Brought my family closer together	0.160	**0.689**	0.229	0.246	0.059	0.140
7. Made me more sensitive to family issues	0.111	**0.787**	0.185	0.107	0.262	0.035
8. Helped me appreciate my family more	0.113	**0.749**	0.271	0.207	0.093	0.135
9. Made me more aware of what my family means to me	0.139	**0.576**	0.371	0.126	0.238	0.117
10. Made me a more effective person	0.143	0.282	**0.681**	0.188	0.092	0.201
11. Taught me how to cope more effectively	0.103	0.335	**0.694**	0.063	0.186	0.180
12. Helped me become a stronger person	0.090	0.296	**0.684**	0.106	0.158	0.107
13. Taught me how I can handle most things	0.012	0.059	**0.814**	0.219	0.193	0.058
14. Led me to deal better with problems	0.093	0.112	**0.780**	0.273	0.117	0.181
15. Helped me to grow emotionally and spiritually	0.076	0.127	**0.593**	0.369	0.214	0.152
16. Helped me become more aware of the support available from others	0.168	0.159	0.397	**0.617**	0.123	0.148
17. Helped me realise who my real friends are	0.178	0.258	0.109	**0.677**	0.190	0.187
18. Led me to feel more positive about others	0.092	0.132	0.254	**0.674**	0.162	0.220
19. Led me to meet people who have become some of my best friends	0.057	0.092	0.187	**0.684**	0.213	0.126
20. Made me more compassionate to those in similar situations	0.160	0.352	0.142	0.393	**0.501**	0.014
21. Made me more sensitive to the needs of others	0.381	0.006	0.079	0.181	**0.548**	0.016
22. Made me care more about others	0.120	0.222	0.207	0.234	**0.652**	0.069
23. Made me closer to people I care about	0.030	0.163	0.183	0.200	**0.725**	0.172
24. Taught me that everyone has a right to be valued	0.063	0.146	0.210	0.060	**0.697**	0.316
25. Led me to place less emphasis on material things	0.121	0.041	0.161	0.106	0.338	**0.539**
26. Led me to live my life more simply	0.182	0.129	0.134	0.170	-0.013	**0.758**
27. Led me to change my priorities in life	0.090	0.084	0.128	0.228	0.110	**0.761**
28. Helped me become more focused on real priorities	0.061	0.166	0.313	0.092	0.350	**0.543**

Notes: Factor 1: acceptance, Factor 2: family bonds, Factor 3: growth, Factor 4: relationships, Factor 5: empathy, Factor 6: reprioritization

**Fig 1:**
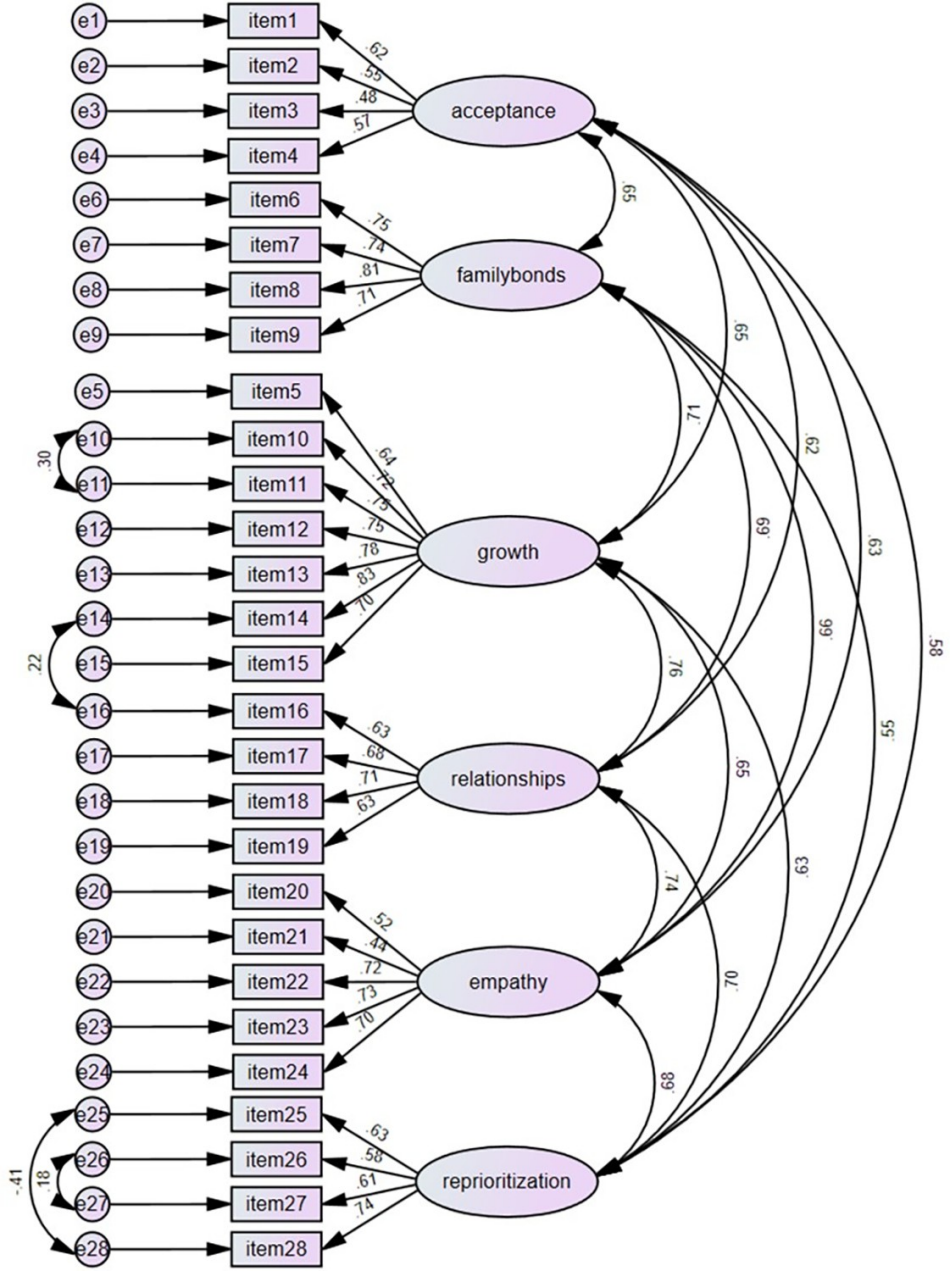
Confirmatory factor analysis for the items of the GBFS (n = 309).

We also performed CFA to examine the construct validity of GBFS in Chinese college students (Fig 1). Results showed that SRMR = 0.032, RMSEA = 0.063, GFI = 0.848, CFI = 0.889, TLI = 0.874, and *χ*^2^/*df =* 2.22 (742.897/335). As the modification indices suggested, four correlation paths between errors were added therefore producing improvements in the model fit: SRMR = 0.031, RMSEA = 0.059, GFI = 0.860, CFI = 0.904, TLI = 0.890, and *χ*^2^/*df =* 2.07 (684.640/331).

#### Concurrent validity

The correlation analyses revealed that the overall score of GBFS negatively correlated with perceived stress (*r* = –0.271, *p*<0.001) but positively related to subjective well-being (*r* = 0.354, *p*<0.001) among Chinese college students, suggesting that the GBFS has good concurrent validity. Regarding the individual dimensions, acceptance, family bonds, growth, and relationships showed statistically significant negative correlations with perceived stress (*r* ranged from –0.375 to –0.155), while empathy and reprioritization showed no statistically significant correlations. Nevertheless, the scores of all dimensions were positively related to subjective well-being, with the correlation coefficients ranging from 0.166 to 0.363 (Table 5).

**Table 5:** Correlations between benefit finding and perceived stress and subjective well-being.

Dimensions	M (SD)	Skewness	Kurtosis	Perceived stress		Subjective well-being	
				*r*	*p*	*r*	*p*
Benefit finding	104.5 (15.35)	–0.418	0.281	–0.271	<0.001	0.354	<0.001
Acceptance	14.4 (2.95)	–0.526	0.536	–0.258	<0.001	0.307	<0.001
Family bonds	15.5 (3.13)	–0.544	0.009	–0.184	0.001	0.232	<0.001
Growth	26.5 (4.94)	–0.752	0.840	–0.375	<0.001	0.363	<0.001
Relationships	14.6 (2.82)	–0.343	0.194	–0.155	0.007	0.267	<0.001
Empathy	19.3 (3.30)	–0.692	0.843	–0.106	0.065	0.166	0.004
Reprioritization	14.1 (2.92)	–0.407	0.225	–0.062	0.285	0.247	<0.001

Notes: M = mean, SD = standard deviation

### Reliability

#### Internal consistency

The Cronbach’s α coefficient of the overall Chinese version of GBFS was 0.93. For the dimensions, Cronbach’s α coefficients were 0.78, 0.86, 0.88, 0.71, 0.77, and 0.70 for acceptance, family bonds, growth, relationships, empathy, and reprioritization, respectively. The composite reliability index for each dimension ranged from 0.71 to 0.86 and the McDonald’s omega reliability of the GBFS was 0.94. These results indicate that the GBFS has an appropriate internal consistency.

#### Test-retest reliability

The test-retest reliability of the Chinese version of GBFS with a two-week interval was 0.82 (*p* <0.001). Also, paired sample *t*-test results showed no statistically significant differences in the total score of GBFS among the 50 college students in phases 3 & 4 (*t* = –1.160, *p* = 0.252), suggesting that the GBFS has an excellent test-retest reliability.

## Discussion

This study aimed to translate the GBFS into a simplified Chinese version and examine the psychometric properties among college students. Doing so is important because it helps expand the available cross-cultural research on the GBFS while also providing a useful tool for the assessment of benefit finding in Chinese-speaking populations. The results of this study demonstrated desirable internal consistency and promising test-retest reliability. Factor analyses showed a six-factor model to be an acceptable fit for the data. In addition, the GBFS correlated significantly negatively with perceived stress and significantly positively with subjective well-being, providing evidence for the concurrent validity. The GBFS is therefore a reliable and valid instrument for Chinese college students to ascertain levels of benefit finding in relation to general life stress.

Similar to the studies carried out in other countries [21, 23, 24], this study found a high Cronbach’s α coefficient for the GBFS in Chinese college students. Moreover, each item of this instrument moderately correlated with the overall score, which is aligned with the work of the original authors [8]. These results have demonstrated a high internal consistency across the whole GBFS. Regarding the test-retest reliability, it was determined with a sample of 50 college students and evinced good stability. The results have reinforced the stability of the GBFS to assess benefit finding in response to general life stress. To the best of our knowledge, this study is the first to examine the test-retest reliability of the GBFS, and the two-week interval which is commonly employed when evaluating test-retest reliability [49, 50] was adopted. In a longitudinal study, Zimmaro et al. found that benefit finding measured by a 14-item Benefit Finding Scale significantly increased at a six-month follow-up among patients with colorectal cancer (*p =* 0.03) [51]. More evidence is needed to verify the stability of the GBFS over time and therefore the temporal changes of benefit finding in response to general life stress could be investigated.

As an expert panel rated, almost all items in the GBFS are quite or highly relevant to the construct of benefit finding; that is, the scale has a sound content validity. Although experts’ feedback may be subjective, we involved experts with different educational backgrounds (both master’s and doctoral degrees) to ensure their representativeness. The construct validity of the Chinese version of GBFS was examined with EFA and CFA. Similar to the findings of two previous studies: one conducted in college students [8] and the other in older adults [20], we found a six-factor model of the Chinese version of GBFS. However, the EFA results suggest that “Taught me to be patient” (item 5) was more relevant to the dimension of “growth” (factor loading = 0.533). The CFA results also demonstrated an acceptable goodness of fit between our data and the revised six-factor model. Personal growth refers broadly to a subset of personality development that relates to the process of becoming better in a personally meaningful way [52], while patience is a personality trait. From this perspective, improved patience can be regarded as personal growth during a difficult time, and classifying item 5 into the dimension of “growth” is reasonable. It is also worth noting that we added four correlation paths between errors, as the modification indices suggested. This seems rational since those items are related to the same topic. Nevertheless, the construct of the GBFS can be further explored in different samples (e.g., clinical patients).

While there is a lack of a gold standard instrument to measure benefit finding in the face of general life stress, related measurements (perceived stress and subjective well-being) were simultaneously investigated to examine the concurrent validity of the GBFS. Our findings have suggested that the GBFS score is significantly negatively related to perceived stress, which is generally in accordance with previous studies conducted in other populations. For example, college students who expressed finding benefits were less likely to experience stress during the COVID-19 pandemic [53, 54]. Among patients with colorectal cancer, it was found that greater benefit finding trended towards an association with lower distress [51]. Cassidy et al. also revealed that benefit finding was negatively associated with student hassles [8]. A potential explanation for this phenomenon is that benefit finding as a coping strategy may be a salient buffer for the effects of stress [3]. In the future, the improvement of benefit finding can be a possible target when designing stress management interventions. Nevertheless. the relationships between benefit finding and perceived stress are suggested to be further examined in the context of general life stress.

On the contrary, the GBFS score significantly positively correlated with subjective well-being in college students. These findings are consistent with previous studies conducted in different populations. As reported, benefit finding positively relates to the levels of mental well-being in older adults [20]. In a cluster randomized controlled trial, Cheng et al. reported that benefit finding intervention could significantly improve the psychological well-being of Alzheimer caregivers [55]. A longitudinal study found that benefit finding positively related to the well-being in cancer survivors and parents [56]. Differently, another study found that the well-being of cancer patients was not associated with benefit finding score; however, changes in benefit finding predicted the well-being one year after surgery [57]. Wepf et al. revealed that benefit finding was associated with better mental well-being directly as well as indirectly via better coping [24], which to some extent can explain the relationship between benefit finding and subjective well-being in the present study. Moreover, benefit finding may help individuals derive meaning during difficult times [58], which in turn boosters their well-being [59].

This study had two limitations. First, the participants in this study were composed of college students recruited through a convenience sampling method. The age of the sample was relatively homogeneous, and the proportion of females was lower than that of the overall country (52.9%) [60]. Thus, it would be advisable to explore the applicability of the Chinese version of GBFS in a more diverse sample, for example, by involving vocational undergraduates and postgraduates. Second, this study examined the concurrent validity of the Chinese version of GBFS by analyzing its correlations with perceived stress and subjective well-being. Longitudinal studies could be conducted to explore the predictive validity of this instrument. Despite these limitations, our study demonstrates that the Chinese version of GBFS is a reliable and valid instrument that can be adopted to evaluate benefit finding in relation to general life stress among college students in mainland China.

## Conclusions

This study translated the GBFS into simplified Chinese and evaluated its psychometric properties among college students in mainland China. Results demonstrate that the Chinese version of GBFS has satisfactory psychometric properties with good reliability and validity when measuring benefit finding in relation to general life stress. Researchers and administrators may use the GBFS as an appropriate and effective instrument to ascertain the levels of benefit finding during difficult times. This assessment can facilitate tailored interventions to manage general life stress and improve subjective well-being.

## Supporting information

S1 File The Chinese version of GBFS. (PDF)

S2 File The Chinese GBFS data. (XLS)
