# Supplementary material for: The Chinese version of the general benefit finding scale (GBFS): Psychometric properties in a sample of college students
Source: PLoS One. 2024 May 7;19(5):e0300064. doi: 10.1371/journal.pone.0300064 (PMC11075837; doi:10.1371/journal.pone.0300064)
Supplement: S1 File — (PDF) [file pone.0300064.s001.pdf]

益处发现量表

请仔细阅读以下条目，根据你处于逆境时的感受选择合适的选项。

	完全不同意	不同意	介于中间	同意	完全同意
1. 让我更接受事物	1	2	3	4	5
2. 教会我如何适应我无法改变的事情	1	2	3	4	5
3. 帮助我顺其自然	1	2	3	4	5
4. 给予我更切实际的期望	1	2	3	4	5
5. 教会我要有耐心	1	2	3	4	5
6. 使我的家庭更加紧密	1	2	3	4	5
7. 让我更加体谅家庭问题	1	2	3	4	5
8. 帮助我更加感激我的家人	1	2	3	4	5
9. 让我更加意识到家庭对我的意义	1	2	3	4	5
10. 让我成为更有效率的人	1	2	3	4	5
11. 教会我如何更有效应对	1	2	3	4	5
12. 帮助我成为一个更坚强的人	1	2	3	4	5
13. 教会我如何处理大多数事情	1	2	3	4	5
14. 让我更好地处理问题	1	2	3	4	5
15. 帮助我在情感和精神上成长	1	2	3	4	5

	完全不同意	不同意	介于中间	同意	完全同意
16. 帮助我更加感受到别人的支持	1	2	3	4	5
17. 帮助我认识到谁是我真正的朋友	1	2	3	4	5
18. 让我对别人更有信心	1	2	3	4	5
19. 让我认识到一些人，后来成为好朋友	1	2	3	4	5
20. 让我对处于类似情况的人更有同情心	1	2	3	4	5
21. 让我对他人的需求更加敏感	1	2	3	4	5
22. 使我更关心他人	1	2	3	4	5
23. 使我和在乎的人更亲近	1	2	3	4	5
24. 教会我每个人都有被重视的权利	1	2	3	4	5
25. 让我不再那么强调物质上的东西	1	2	3	4	5
26. 让我的生活更简单	1	2	3	4	5
27. 让我改变了生活中的优先顺序	1	2	3	4	5
28. 帮助我更专注于真正的优先事项	1	2	3	4	5
